# Bridged-Bicyclic Fluorophores Push Photophysical Boundaries for Live-Cell Imaging

**DOI:** 10.1021/cbmi.5c00078

**Published:** 2025-06-27

**Authors:** Dongjuan Si, Lu Wang

**Affiliations:** MOE Key Laboratory of Smart Drug Delivery, MOE Innovative Center for New Drug Development of Immune Inflammatory Diseases, School of Pharmacy, Fudan University, Shanghai 201203, China

Over the past three decades, fluorescence imaging has evolved from a descriptive tool into a precision instrument for dissecting biology at the molecular scale.^1^ This transformation has been propelled by parallel advances in optical instrumentation, such as super resolution microscopy^2–4^ and single molecule tracking,^5^ and in the design of functional small molecule fluorophores. With the latest imaging technologies achieving near-molecular spatial resolution and submillisecond temporal precision, we are approaching the long-sought goal of interrogating biological systems at the molecular level in real time.^6^

While breakthroughs in instrumentation such as stimulated emission depletion (STED) microscopy, structured illumination microscopy (SIM), and single molecule localization microscopy (SMLM) have redefined the optical limits of biological imaging, the performance of these techniques critically depends on the availability of optimized fluorescent probes.^7^ Compared to fluorescent proteins such as green fluorescent protein (GFP), synthetic small molecule dyes offer distinct advantages, including higher brightness, superior photostability, tunable spectral properties, and the capacity for orthogonal labeling.^8,9^ When combined with compatible protein or RNA tags, these dyes enable high speed, high resolution, and multiplexed imaging in live-cell nanoscopy.^10,11^

A central driver of fluorophore innovation is the rational design of auxochromes, which modulate the electronic, photophysical, and chemical properties of the dye core. Successive generations of auxochrome-modified fluorophores have addressed specific performance limitations, from sulfonated derivatives such as Alexa Fluor^12^ to azetidine substituted Janelia Fluor (JF) dyes,^13^ and to deuterated, hydrophilic,^14^ or sulfamide modified MaP dyes.^15^ While each class improves aspects such as brightness, photostability, or aqueous compatibility, no single platform has yet achieved an optimal combination of quantum yield, photostability, hydrophilicity, synthetic accessibility, and biological versatility in a unified molecular scaffold.

In a recent *Nature Methods* study, Chen et al. introduce a compelling solution: bridged bicyclic dyes (BDs) featuring SO_2_ or O substituted azabicyclo[3.2.1]­octane auxochromes, a chemically rigid, electronically tunable motif that simultaneously enhances quantum yield, mitigates nonradiative decay, and improves aqueous solubility. This strategic molecular architecture enables a new class of fluorophores that span the UV to visible spectrum with minimal compromise on performance^16^ (Figure 1a,b). When conjugated to HaloTag ligands, BD dyes function as high performance chemogenetic reporters, enabling rapid and specific labeling in both in vitro and in vivo contexts.

**Figure 1:**
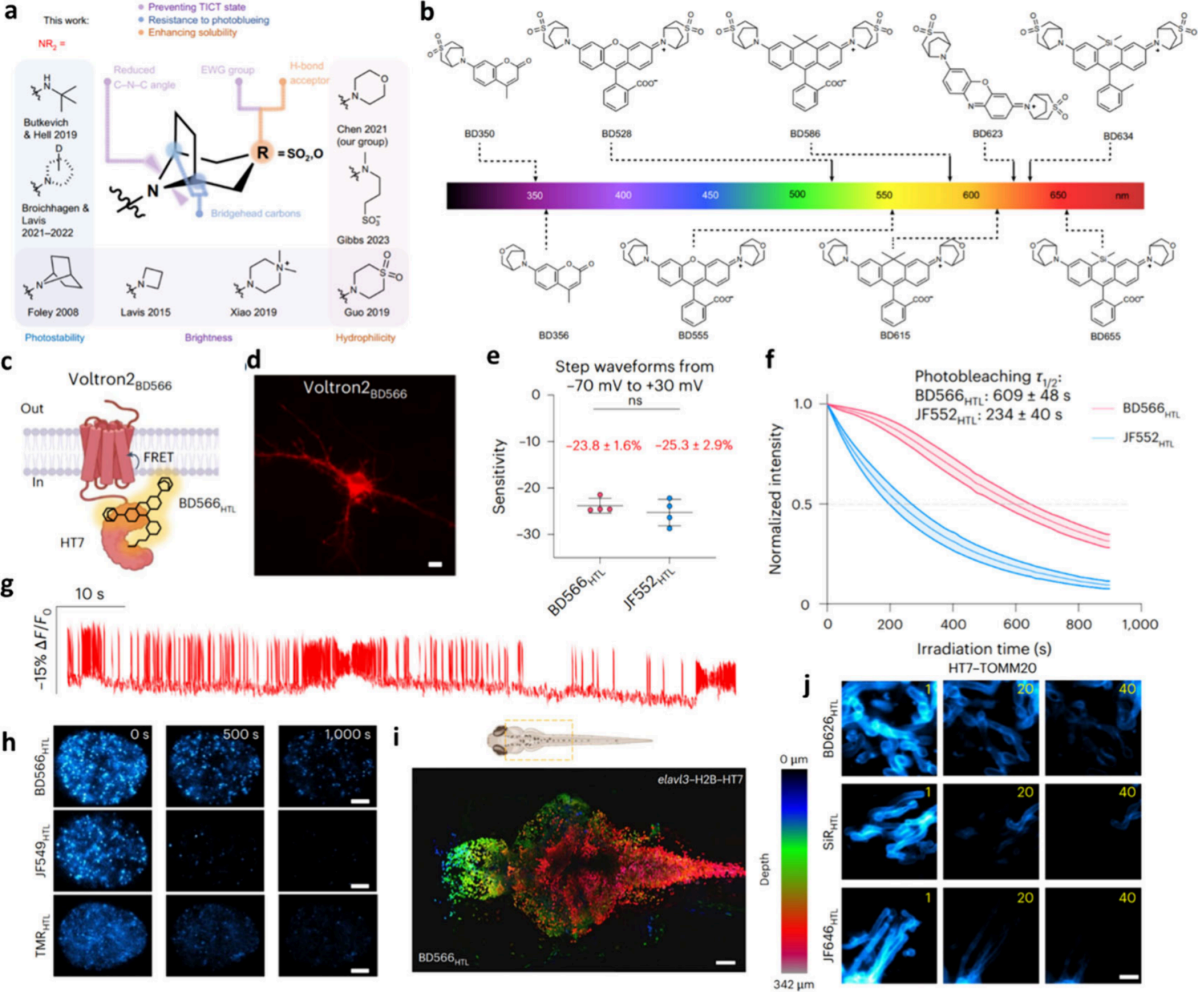
BD_HTLs_ as versatile tools for general imaging in cells and animals. (a) Strategies for development of fluorophores with bridged bicyclic auxochromes. (b) Structures of the BD dye palette. (c–g) Time-lapse confocal imaging of neuronal voltage dynamics comparing the voltage sensitivities of BD566HTL and JF552HTL on Voltron2 expressed in HEK293T cells. Scale bar, 10 μm. (h) Indicated time points during continuous time-lapse single-molecule imaging of fixed U2OS cells stably expressing H2B-HaloTag7. Scale bar, 5 μm. (i) *Z*-stack confocal images of live zebrafishes expressing *elavl3*-H2B-HT7 and labeled with BD566_HTL_ or BD626_HTL_. Scale bar, 30 μm. (j) Time-lapse STED images of live HeLa cells expressing TOMM20-HT7 labeled with BD626_HTL_, SiR_HTL_, and JF646_HTL_. Scale bars, 1 μm. Reproduced with permission from ref 16. Copyright 2025 Springer Nature.

What sets the BD dyes apart is their unique convergence of photophysical excellence and biological utility. In side-by-side comparisons with state-of-the-art fluorophores such as JF549 and TMR, BD566_HTL_, when integrated with the Voltron2 hybrid voltage sensor, enabled bright, photostable, and high-sensitivity functional voltage imaging, thereby providing a robust platform for high-speed, time-resolved monitoring of neuronal activity (Figure 1c–g). In addition, BD dyes demonstrated significantly enhanced signal-to-noise ratios, with up to a 2.8-fold increase in single-molecule tracking brightness and a 4.3-fold prolongation of track durations, highlighting their distinct advantages in single-molecule imaging (Figure 1h).

Crucially, the BD dye platform demonstrated broad adaptability across imaging modalities and biological systems. In zebrafish embryos, BD dyes enabled bright and specific labeling of developing neural structures, allowing high resolution 3D reconstructions of the nervous system (Figure 1i). In STED imaging, BD626_HTL_ maintained superior fluorescence retention under high intensity illumination- three times higher than that of benchmark dyes (Figure 1j). In plant cells imaged with SIM, BD626_HTL_ outperformed JF646_HTL_ by nearly 20-fold in photostability, affirming its robustness across kingdoms and cell types.

The BD platform sets a new benchmark by resolving longstanding trade-offs among brightness, stability, hydrophilicity, and biological compatibility. Its modular structure offers a fertile foundation for further derivatization, enabling future adaptation to emerging imaging demands such as deep tissue volumetric imaging, high speed biosensing, and in vivo functional interrogation.

In conclusion, bridged bicyclic fluorophores signal a new era in chemical probe design. By unifying electronic precision with structural rigidity and biological versatility, they provide a next generation toolkit for bioimaging—one that meets the stringent requirements of modern microscopy while remaining accessible to the broader biological community. As biological questions grow more complex and imaging demands more exacting, innovations like BD dyes will be pivotal in driving both technological capability and biological discovery forward.
